# Oncogenic KRAS modulates mitochondrial metabolism in human colon cancer cells by inducing HIF-1α and HIF-2α target genes

**DOI:** 10.1186/1476-4598-9-293

**Published:** 2010-11-13

**Authors:** Sang Y Chun, Craig Johnson, Joseph G Washburn, Marcia R Cruz-Correa, Duyen T Dang, Long H Dang

**Affiliations:** 1 University of Michigan Comprehensive Cancer Center, Ann Arbor, MI, USA; 2 The University of Michigan Comprehensive Cancer Center Affymetrix and cDNA Microarray Core Facility, Ann Arbor, MI, USA; 3 The University of Puerto Rico Cancer Center, San Juan, PR, USA; 4 Division of Gastroenterology, Department of Internal Medicine, University of Michigan, Ann Arbor, MI, USA; 5 Division of Hematology/Oncology, Department of Internal Medicine, University of Florida Shands Cancer Center, University of Florida, Gainesville, FL, USA

## Abstract

**Background:**

Activating *KRAS *mutations are important for cancer initiation and progression; and have recently been shown to cause primary resistance to therapies targeting the epidermal growth factor receptor. Therefore, strategies are currently in development to overcome treatment resistance due to oncogenic KRAS. The hypoxia-inducible factors-1α and -2α (HIF-1α and HIF-2α) are activated in cancer due to dysregulated ras signaling.

**Methods:**

To understand the individual and combined roles of HIF-1α and HIF-2α in cancer metabolism and oncogenic KRAS signaling, we used targeted homologous recombination to disrupt the oncogenic *KRAS*, *HIF-1α*, and *HIF-2α *gene loci in HCT116 colon cancer cells to generate isogenic HCT116^WT KRAS^, HCT116^HIF-1α-/-^, HCT116^HIF-2α-/-^, and HCT116^HIF-1α-/-HIF-2α-/- ^cell lines.

**Results:**

Global gene expression analyses of these cell lines reveal that HIF-1α and HIF-2α work together to modulate cancer metabolism and regulate genes signature overlapping with oncogenic KRAS. Cancer cells with disruption of both *HIF-1α *and *HIF-2α *or oncogenic *KRAS *showed decreased aerobic respiration and ATP production, with increased ROS generation.

**Conclusion:**

Our findings suggest novel strategies for treating tumors with oncogenic *KRAS *mutations.

## Introduction

Oncogenic *ras *mutations (involving *HRAS*, *NRAS*, and *KRAS *genes) are found in approximately 30% of all human tumors; with mutations affecting *KRAS *being the most prevalent. *KRAS *mutations are most prevalent in pancreatic (72-90%), thyroid (55%), colorectal (32-57%), and lung cancers (15-50%) [1,2,1,2]. Point mutations at codons 12, 13, or 61 result in stabilization of KRAS in the GTP-bound conformation, rendering it constitutively active [3]. Activated ras signaling contributes to oncogenic transformation by providing molecular signals that promote cell proliferation, obstruct cell death, inhibit cellular differentiation, and induce angiogenesis [4]. Underlying these cellular processes, ras transformed cells also undergo significant metabolic adaptation [5].

The hypoxia-inducible factors-1α and -2α (HIF-1α and HIF-2α) are transcription factors that are overexpressed in cancer and linked to cancer progression [6,7]. Structurally, HIF-1α and HIF-2α are partially related, sharing 48% overall amino acid identity and two identical proline residues in their oxygen-dependent degradation domains [8,9]. HIF-1α and HIF-2α dimerize with HIF-1β to form HIF-1 and HIF-2, respectively. HIF-1α and HIF-2α overexpression are driven by intratumoral hypoxia, growth factor signaling, and genetic mutations in oncogenes and tumor suppressor genes [10,11]. Under normoxia, HIF-1α and HIF-2α are ubiquitinated through an oxygen-dependent interaction with the von Hippel-Lindau protein (pVHL) and degraded by the 26S proteasome [12,13]. Under hypoxic conditions, HIF-1α and HIF-2α proteins accumulate, translocate to the nucleus, dimerize with HIF-1β, and transactivate target genes. In cancer, genetic alterations in tumor suppressor genes and oncogenes also induce HIF-1α and HIF-2α overexpression, and lead to the transactivation of target genes. MAPK signaling downstream of ras has been shown to lead to the phosphorylation of HIF-1α and, thereby, stimulate its transcriptional activity [11,14].

Both HIF-1α and HIF-2α induce the expression of target genes important for tumor angiogenesis, cell growth and survival, and metastasis [7,15,16]. To date, regulation of cancer glucose metabolism has been predominantly linked to HIF-1α rather than HIF-2α. HIF-1α induces the expression of glucose transporters and glycolytic enzymes that promote glucose uptake and glycolysis [17,18]. This has been well demonstrated under hypoxic conditions; and more recently under normoxic conditions [10,19,20]. HIF-1α was also recently shown to induce the expression of pyruvate dehydrogenase kinase 1 (PDK1) under hypoxic conditions [21,22]. PDK1 is a kinase that inhibits pyruvate dehydrogenase (PDH), an enzyme that catalyzes the conversion of pyruvate to acetyl-CoA. This leads to suppression of pyruvate entry into the TCA cycle, with consequent suppression of mitochondrial oxygen consumption. Through these mechanisms, HIF-1α is thought to mediate aerobic glycolysis and contributes to carcinogenesis.

Furthermore, both HIF-1α and HIF-2α were shown to regulate the exchange of COX4 (cytochrome *c *oxidase 4) subunits under hypoxic conditions; thereby increasing mitochondrial respiration efficiency and decreasing ROS production [23]. These findings implicate HIF-1α and HIF-2α in balancing glycolysis and aerobic respiration to maintain ATP production and prevent toxic ROS generation [23].

To understand the individual and combined roles of HIF-1α and HIF-2α in cancer metabolism and oncogenic KRAS signaling, we used targeted homologous recombination to disrupt the oncogenic *KRAS*, *HIF-1α*, and *HIF-2α *gene loci in HCT116 colon cancer cells to generate isogenic HCT116^WT KRAS^, HCT116^HIF-1α-/-^, HCT116^HIF-2α-/-^, and HCT116^HIF-1α-/-HIF-2α-/- ^cell lines. These cell lines are then subjected to global gene expression analyses. We characterized the metabolic adaptation mediated by oncogenic KRAS and both HIF-1α and HIF-2α.

## Results

### The metabolic transcriptomes regulated by oncogenic KRAS and by both HIF-1α and HIF-2α show significant overlap

HCT116 cell line with targeted disruption of the oncogenic *KRAS *allele was generated by targeting exon 2 for homologous recombination, as previously described [20]. This resulted in HCT116 cells with just the wild-type allele (HCT116^WT KRAS^), as confirmed by genomic DNA sequencing of these cell lines (Additional file 1, Fig. S1). The *HIF-1α *gene locus was disrupted by targeting exons 3 and 4; and the *HIF-2α *gene locus was disrupted by targeting exons 5 and 6 for homologous recombination, as previously described [20]. Loss of HIF-1α, HIF-2α, and both HIF-1α and HIF-2α proteins in the resultant isogenic knockout cell lines (HCT116^HIF-1α-/-^, HCT116^HIF-2α-/-^, and HCT116^HIF-1α-/-HIF-2α-/-^) were confirmed by Western blot analysis (Additional file 2, Fig. S2).

**Figure 1:**
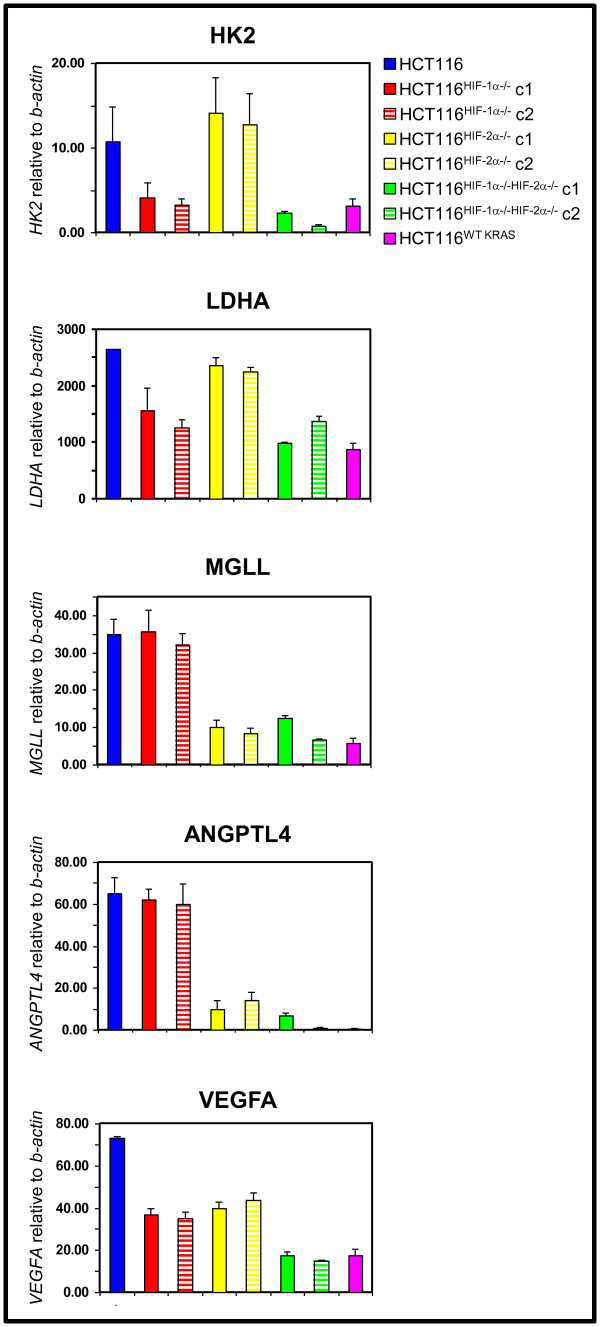
**Analyses of genes regulated by oncogenic KRAS, HIF-1α, HIF-2α, and both HIF-1α and HIF-2α**. Expression of target genes in HCT116, HCT116^HIF-1α-/-^, HCT116^HIF-2α-/-^, HCT116^HIF-1α-/-HIF-2α-/-^, and HCT116^WT KRAS ^cell lines. *HK2*, hexokinase 2; *LDHA*, lactate dehydrogenase A; *MGLL*, monoglyceride lipase; *ANGPTL4*, angiopoietin-like protein 4; and *VEGFA*, vascular endothelial growth factor A relative to *β-actin *were measured by real-time reverse transcription-PCR (n = 5). Bars, stdev. p < 0.05 by Student's t test comparing knockout cells with parental HCT116 cells. c, clone.

To systematically identify genes that are regulated by HIF-1α, HIF-2α, both HIF-1α and HIF-2α, and oncogenic KRAS we performed global gene expression analyses on HCT116, HCT116^HIF-1α-/-^, HCT116^HIF-2α-/-^, HCT116^HIF-1α-/-HIF-2α-/-^, and HCT116^WT KRAS ^cells. Using a cut-off of > 2.0-fold difference in gene expression between HCT116 versus HCT116^HIF-1α-/-HIF-2α-/- ^cells, we identified genes regulated exclusively by HIF-1α or HIF-2α, and by both HIF-1α and HIF-2α. The expression of some of these genes was confirmed by real-time RT-PCR (Figure 1). Consistent with the literature, some genes were exclusively HIF-1α targets, including those involved in glycolysis (*HK2*, *LDHA*); some genes were exclusively HIF-2α targets, i.e. *ANGPTL4 *and *MGLL*; and some genes were HIF-1α and HIF-2α co-regulated targets, i.e. *VEGFA *(Figure 1). To determine whether HIF-1α and HIF-2α target genes are also downstream targets of oncogenic KRAS, we compared the gene sets regulated by HIF-1α, HIF-2α, and by both HIF-1α and HIF-2α with the gene set regulated by oncogenic KRAS (HCT116 versus HCT116^WT KRAS^). We found that oncogenic KRAS regulates genes that were induced by HIF-1α alone, HIF-2α alone, or by both HIF-1α and HIF-2α (Figure 1).

**Figure 2:**
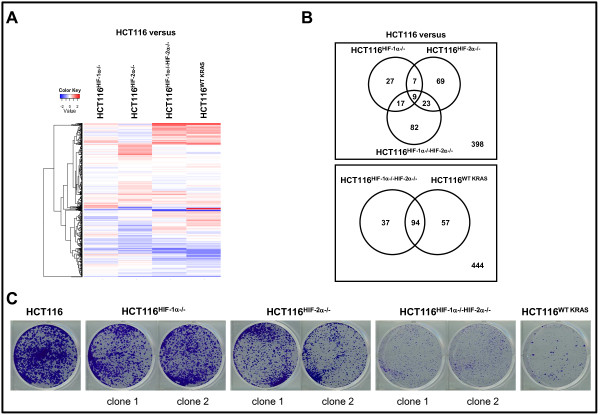
**Analyses of metabolism gene set regulated by oncogenic KRAS and HIF**. **A**, heatmap analysis comparing the following metabolism gene sets: HCT116 vs HCT116^HIF-1α-/-^, HCT116 vs HCT116^HIF-2α-/-^, HCT116 vs HCT116^HIF-1α-/-HIF-2α-/-^, and HCT116 vs HCT116^WT KRAS ^cells. Expression values are averages of three samples. **B**, Venn diagrams analyzing the extent of overlap of the following metabolism gene sets: 1) HCT116 vs HCT116^HIF-1α-/-^, HCT116 vs HCT116^HIF-2α-/-^, and HCT116 vs HCT116^HIF-1α-/-HIF-2α-/-^; 2) HCT116 vs HCT116^HIF-1α-/-HIF-2α-/- ^and HCT116 vs HCT116^WT KRAS^. Each circle represents a single gene set; and the number of genes common between the gene sets is denoted within the overlaps of the circles. **C**, Clonogenic survival assay of HCT116, HCT116^HIF-1α-/-^, HCT116^HIF-2α-/-^, HCT116^HIF-1α-/-HIF-2α-/-^, and HCT116^WT KRAS ^cells. c, clone.

To focus on the regulation of genes controlling nutrients metabolism, we performed a heatmap analysis of genes categorized by the "GO" term "metabolism" (Figure 2A). The gene terms and fold changes are shown in Table 1. Using the color *blue *for negatively and *red *for positively regulated genes and data analysis using Venn diagrams, we made the following observations. First, HIF-1α and HIF-2α regulate both overlapping and unique metabolism genes (Figure 2A-B). To our surprise, the absence of HIF-2α led to a greater change in the number of affected metabolism genes than the absence of HIF-1α (Figure 2B). The absence of both HIF-1α and HIF-2α led to an increase in both the number and change in expression levels of affected genes (Figure 2A-B). Second, the metabolic gene set regulated by oncogenic KRAS showed significant overlap with the metabolic gene set regulated by both HIF-1α and HIF-2α (HCT116 versus HCT116^WT KRAS ^in comparison to HCT116 versus HCT116^HIF-1α-/-HIF-2α-/-^) (Figure 2A-B). This similarity in the metabolic gene sets reflects the similar extent of suppression of clonogenic survival upon loss of oncogenic KRAS or both HIF-1α and HIF-2α (Figure 2C).

**Table 1:** Fold change in expression of metabolism genes

HCT116 versus			
**Gene symbol**	**HCT116**^**HIF-1α-/-**^	**HCT116**^**HIF-2α-/-**^	**HCT116**^**HIF-1α-/-HIF-2α-/-**^

ACAT2	1.435	1.346	3.037
GSTP1	1.416	1.257	2.552
G6PD	1.068	1.144	5.369
METTL7B	1.723	0.758	2.947
GSTM4	1.519	0.667	2.643
NAT1	1.799	0.857	2.513
SULF2	1.047	0.584	3.444
UGT8	0.833	0.543	2.312
PCAF	1.131	0.617	3.6
ABP1	1.17	1.524	7.443
ALPP	1.193	2.608	5.763
METTL7A	0.831	1.017	2.359
MDH1B	0.915	0.943	2.237
MAN1C1	1.333	0.775	2.076
CRYZL1	1.214	0.756	2.252
LSS	1.103	0.898	2.052
HIBCH	1.021	0.757	2.094
SRR	0.988	0.598	2.04
ZADH2	0.665	0.725	2.381
TP53I3	1.116	1.06	3.215
ALPPL2	0.887	1.537	3.467
ALDH7A1	0.985	1.215	2.211
ACAA2	1.1	1.466	2.539
DHRS2	5.301	0.304	2.496
DHRS3	0.492	0.515	0.306
DPYD	0.445	0.268	0.251
ASNS	0.646	0.816	0.126
BCKDHA	0.544	0.944	0.315
PCK2	0.661	0.798	0.214
TMEM68	0.849	1.18	0.38
ALDOC	0.62	1.178	0.301
PSAT1	0.787	0.98	0.257
ATP9A	0.71	0.445	0.257
RDHE2	0.841	0.405	0.443
MOCOS	0.797	0.419	0.361
PHGDH	0.391	0.738	0.321
ACSM3	0.448	0.512	0.488
ACSS2	0.603	0.717	0.371
MTHFD1L	0.574	0.99	0.378
GAA	0.726	0.474	0.464
GFPT1	0.793	0.644	0.499
UAP1	0.99	0.886	0.406
AGPAT7	1.028	0.757	0.428
PLD3	0.96	0.651	0.453
PLA2G4C	1.301	0.608	0.37
LIAS	1.213	0.897	0.493
PYCR1	1.005	0.76	0.444
MECR	0.858	0.766	0.494
ACSL5	0.228	0.334	0.055
MMP1	0.395	0.07	0.059

Note: expression values are normalized to those in HCT116 cells.

### Absence of oncogenic KRAS or both HIF-1α and HIF-2α reduced the expression of enzymes regulating essential steps in mitochondrial phospholipids synthesis

**Figure 3:**
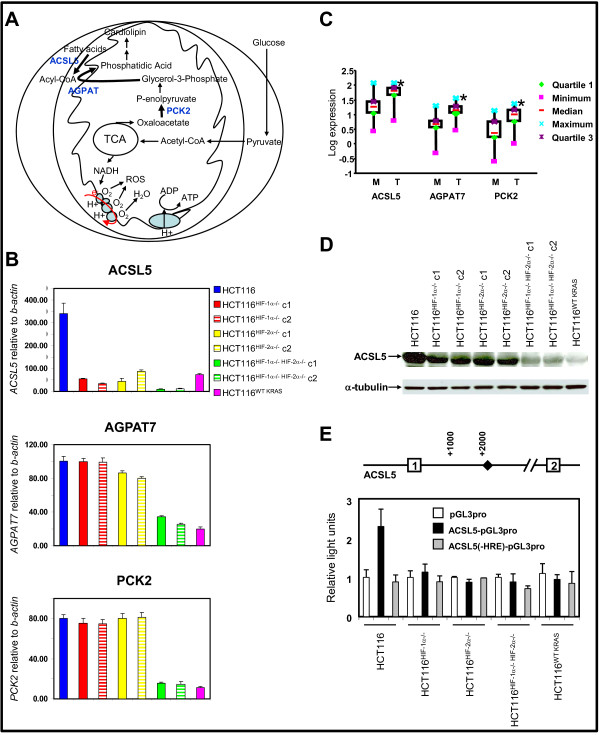
**Analyses of target genes regulating phospholipids synthesis**. **A**, overview of mitochondrial respiration and the contributory role of mitochondrial phospholipids synthesis. **B**, expression of genes regulating phospholipids synthesis in HCT116, HCT116^HIF-1α-/-^, HCT116^HIF-2α-/-^, HCT116^HIF-1α-/-HIF-2α-/-^, and HCT116^WT KRAS ^cell lines. c, clone. *ACSL5*, acyl-CoA synthetase 5; *AGPAT7*, 1-acyl-sn-glycerol-3-phosphate acyltransferase 7; and *PCK2*, phosphoenolpyruvate carboxykinase 2 relative to *β-actin *were measured by real-time reverse transcription-PCR (n = 5). Bars, stdev. p < 0.05 by Student's t test comparing knockout cells with parental HCT116 cells. **C**, Expression of *ACSL5, AGPAT7, and PCK2 *in primary colon cancers. Gene expression, relative to *β-ACTIN*, in normal mucosa and primary tumor was determined by real-time RT-PCR. Log expression values were graphed as box and whiskers plots; showing median expression (horizontal line), surrounded by the first and third quartiles of those values (box), and the extreme values (whiskers). Hypothesis testing was performed using the Wilcoxon signed-rank test, with * = p < 0.05 considered statistically significant, comparing tumor to mucosa. **D**, western blot of ACSL5 in cell lines, with antibody to α-tubulin as loading control. c, clone. **E**, ACSL5 promoter activity in HCT116, HCT116^HIF-1α-/-^, HCT116^HIF-2α-/-^, and HCT116^HIF-1α-/-HIF-2α-/- ^cells (n = 5). Diamond represents HRE site. The pGL3pro construct is a minimal promoter Firefly luciferase reporter. The ACSL5-pGL3pro construct contains an 1883 bp fragment of the 5' untranslated region of the *ACSL5 *gene with the HRE site. This construct is subjected to site-directed mutagenesis at the HRE site to generate the ACSL5(-HRE)-pGL3pro. Bars, stdev. p < 0.05 by Student's t test comparing transfection with pGL3pro construct versus ACSL5-pGL3pro or ACSL5(-HRE)-pGL3pro constructs.

Combing through the gene expression data for groups of genes participating in the same metabolic pathway, we identified three genes regulated by oncogenic KRAS and by both HIF-1α and HIF-2α: ACSL5, PCK2, and AGPAT7 (Table 1). These genes encode for enzymes that catalyze rate-limiting steps in phospholipids biosynthesis (Figure 3A). PCK2, or phosphoenolpyruvate carboxykinase 2, is a mitochondrial enzyme that catalyzes the conversion of oxaloacetate to phosphoenolpyruvate in the mitochondria, a rate-limiting step in glyceroneogenesis. ACSL5, or acyl-CoA synthetase 5, is a mitochondrial enzyme that catalyzes the formation of acyl-CoA, which together with glycerol-3-phosphate forms essential intermediates for phospholipids synthesis. ACSL5 has been shown to induce the synthesis of the mitochondrial phospholipid, cardiolipin [24]. Based on sequence homology to other members of the 1-acyl-sn-glycerol-3-phosphate acyltransferase (AGPAT), AGPAT7 is thought to participate in the conversion of lysophosphatidic acid to phosphatidic acid in phospholipids biosynthesis. However, the specific function of AGPAT7 and its subcellular localization have not been defined.

The expression of all three genes in HCT116 cells was confirmed by real-time RT-PCR (Figure 3B). They are induced by oncogenic KRAS and also required the presence of both HIF-1α and HIF-2α for maximal induction (Figure 3B). To further determine the relevance of these genes to the pathogenesis of colorectal cancer, we compared their expression in primary colorectal cancer specimens and matched normal mucosa (Figure 3C). All three genes were significantly induced in tumors compared to normal mucosa (Figure 3C).

This data suggested an as yet unidentified role for oncogenic KRAS, HIF-1α, and HIF-2α in mitochondrial phospholipids synthesis. To determine how mitochondrial cardiolipin synthesis is regulated, we went on and further characterize ACSL5. Similar to ACSL5 mRNA level, the absence of either HIF-1α or HIF-2α led to decrease in ACSL5 protein level; and the absence of both HIF-1α and HIF-2α or oncogenic KRAS led to the most significant reduction (Figure 3B and 3D).

We next asked whether HIF-1α and HIF-2α regulate the ACSL5 promoter. Examination of the 5'-flanking sequence of the *ACSL5 *gene, using TFSEARCH engine, revealed a hypoxia response element (HRE; RCGTG, where R is A or G) indicating a putative HIF-α binding site (Figure 3E). To determine if HIF-1α and HIF-2α transactivate the ACSL5 promoter, we measured ACSL5 promoter activity using dual-luciferase assays. An 1883 bp fragment derived from the 5' untranslated region of the *ACSL5 *gene, containing the HRE site, was linked to a minimal promoter Firefly luciferase reporter construct (pGL3pro) to generate the ACSL5-pGL3pro construct. To determine the role of HIF-1α and HIF-2α binding, we performed site-directed mutagenesis at the HRE site to generate the construct ACSL5(-HRE)-pGL3pro. These three constructs (pGL3pro, ACSL5-pGL3pro, and ACSL5(-HRE)-pGL3pro) were separately co-transfected with CMV-Renilla luciferase into HCT116, HCT116^*HIF-1α-/-*^, HCT116^*HIF-2α-/-*^, HCT116^*HIF-1α-/-HIF-2α-/-*^, and HCT116^WT KRAS ^cells (Figure 3E). In HCT116 cells, transfection with ACSL5-pGL3pro led to ~2.3-fold induction in Firefly luciferase activity in comparison to transfection with either pGL3pro or ACSL5(-HRE)-pGL3pro (Figure 3E). The absence of HIF-1α, HIF-2α, both HIF-1α and HIF-2α, or oncogenic KRAS led to an almost complete suppression of Firefly luciferase activity (Figure 3E). Altogether, these data suggest that HIF-1α and HIF-2α directly transactivate the ACSL5 promoter at the HRE site.

### Absence of oncogenic KRAS or both HIF-1α and HIF-2α led to decreased cardiolipin level and inefficient mitochondrial respiration

We have identified three enzymes important for early steps in phospholipids synthesis (ACSL5, PCK2, and AGPAT7) whose coordinated expressions are induced by oncogenic KRAS, and by HIF-1α and HIF-2α. ACSL5 has been shown to induce the synthesis of the mitochondrial phospholipid, cardiolipin [24].

**Figure 4:**
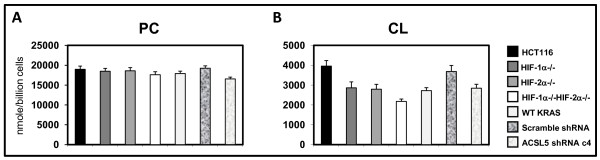
**Quantitation of total cellular (A) phosphatidyl choline (PC) and (B) cardiolipin (CL) levels in HCT116, HCT116^HIF-1α-/-HIF-2α-/-^, HCT116^WT KRAS ^cells, HCT116 cells transduced with control scramble shRNA, and HCT116 cells transduced with ACSL5 shRNA (n = 3)**. Bars, stdev. p < 0.05 by Student's t test comparing knockout cells or shRNA transduced cells with parental HCT116 cells. Four lentiviral constructs carrying ACSL5 shRNA were tested for effectiveness in suppressing ACSL5 expression in comparison to the lentiviral construct carrying control scramble shRNA (Additional file 3, Fig. S3). Clone H45551 was chosen for subsequent experiments as it was the most effective (Additional file 3, Fig. S3).

Cardiolipin is an important component of the inner mitochondrial membrane, where it constitutes about 20% of the total lipid. Cardiolipin serves as an insulator by interacting with electron transport chain proteins to optimize respiration. Mitochondria deficient in cardiolipin malfunction, which result in decreased ATP production and increased ROS generation [25,26]. We next asked whether total cellular phosphatidyl choline (PC) and cardiolipin (CL) levels are affected by the expression of oncogenic KRAS, and HIF-1α and HIF-2α. We compared the following sets of cell lines: HCT116, HCT116^*HIF-1α-/-*^, HCT116^*HIF-2α-/-*^, HCT116^HIF-1α-/-HIF-2α-/-^, and HCT116^WT KRAS ^cells. Phospholipids species were measured in collaboration with Lipomics Technology Inc. We found that CL level was significantly decreased in the absence of HIF-1α, HIF-2α, or oncogenic KRAS; and was most decreased in the absence of both HIF-1α and HIF-2α (Figure 4). PC level was also decreased but to a lesser extent.

**Figure 5:**
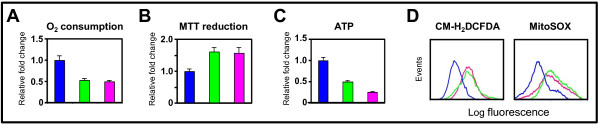
**Effects of genetic inactivation of oncogenic *KRAS *or both *HIF-1α *and *HIF-2α *on mitochondrial metabolism**. **A**, change in cellular O_2 _consumption; **B**, change in MTT reduction; and **C**, change in intracellular ATP level in HCT116^HIF-1α-/-HIF-2α-/- ^and HCT116^WT KRAS ^relative to HCT116 cells (n = 5). Bars, stdev. p < 0.05 by Student's t test comparing knockout cells with parental HCT116 cells. **D**, cellular and mitochondrial ROS levels in HCT116, HCT116^HIF-1α-/-HIF-2α-/-^, and HCT116^WT KRAS ^cells, as measured by the fluorescent dyes CM-H_2_DCFDA and MitoSOX.

To determine effects on mitochondrial respiratory function, we first measured cellular oxygen (O_2_) consumption; as mitochondrial respiration is the main source for cellular O_2 _consumption. We found that loss of both HIF-1α and HIF-2α or oncogenic KRAS significantly decreased cellular O_2 _consumption (Figure 5A).

Decrease in mitochondrial respiration can result from either decreased flux through the TCA cycle or less efficient mitochondrial respiration. We began to address these two possibilities by measuring TCA cycle activity and mitochondrial respiration efficiency.

To determine TCA cycle activity, we measured the activity of one of the TCA cycle enzymes, succinate dehydrogenase, which can be measured by the reduction of methylthiazoletetrazolium (MTT) dye [27]. We found that loss of both HIF-1α and HIF-2α or oncogenic KRAS increased MTT reduction (Figure 5B). Our result suggests that the decrease in O_2 _consumption upon loss of both HIF-1α and HIF-2α or oncogenic KRAS is not due to decreased flux through the TCA cycle; in fact, there is increased flux through the TCA cycle. Thus, the decrease in mitochondrial respiration might be secondary to decreased mitochondrial respiration efficiency.

Mitochondrial respiration efficiency is dependent on the efficiency of the electron transport chain. The mitochondrial respiratory chain consists of four complexes (I-IV). Electrons are transferred through a series of acceptor cytochromes in complexes I-III. At complex IV, O_2 _serves as the final electron acceptor, and is reduced to water. At complexes I, III, and IV, protons are pumped outward across the inner mitochondrial membrane, thus creating an electrochemical gradient with negative charges inside the mitochondrial matrix. The electrochemical gradient generated is then efficiently coupled to ATP production by the inward flow of protons at the F_1_F_0_ATP synthase complex. Inefficient electron transfers through the electron transport chain may lead to early transfer of electrons to O_2 _at complexes I and III and form ROS. Dysfunction at complex IV may lead to inability to couple the electrochemical gradient with ATP production. Thus, measurements of cellular ATP level and ROS production would reflect the efficiency of mitochondrial respiration. We found that loss of both HIF-1α and HIF-2α or oncogenic KRAS in HCT116 cells led to decreased ATP level, and increased cellular and mitochondrial ROS levels (Figure 5C and 5D). Quantitation of the flow cytometry tracings shows 2.1-fold and 2.1-fold increases in total cellular ROS; and 1.9-fold and 1.8-fold increases in mitochondrial ROS upon loss of both HIF-1α and HIF-2α, and oncogenic KRAS, respectively. Altogether, these data show that the presence of oncogenic KRAS and both HIF-1α and HIF-2α in cancer cells lead to increased cardiolipin level and enhanced mitochondrial respiration efficiency.

### Induction of ACSL5 is directly responsible for maintaining cardiolipin level and efficient mitochondrial respiration in cancer

**Figure 6:**
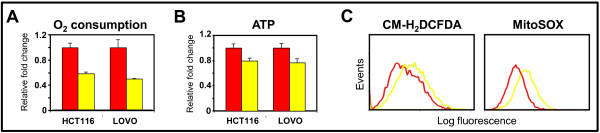
**Effects of ACSL5 gene knockdown on mitochondrial metabolism and tumorigenesis**. **A**, change in cellular O_2 _consumption; and **B**, change in intracellular ATP level in HCT116 and LOVO cells transduced with ACSL5 shRNA relative to control scramble shRNA (n = 5). Bars, stdev. p < 0.05 by Student's t test comparing cells transduced with ACSL5 shRNA versus control scramble shRNA. **C**, cellular and mitochondrial ROS levels in HCT116 cells transduced with ACSL5 shRNA versus control scramble shRNA, as measured by the fluorescent dyes CM-H_2_DCFDA and MitoSOX.

We next asked whether the induction of ACSL5 directly contributes to the increase in cellular phospholipids levels and efficient mitochondrial respiration. We suppressed ACSL5 expression in HCT116 and LOVO human colon cancer cells using lentiviral shRNA (Additional file 3, Fig. S3). In comparison to transduction with lentivirus carrying control scramble shRNA, transduction by lentivirus carrying ACSL5 shRNA led to decreased PC and CL levels (Figure 4). Compared to transduction with lentivirus carrying control scramble shRNA, transduction by lentivirus carrying ACSL5 shRNA also led to decreased cellular O_2 _consumption, decreased ATP production, and increased ROS production (Figure 6A-C). Quantitation of the flow cytometry tracings shows 1.5-fold and 1.5-fold increases in total cellular ROS and mitochondrial ROS upon suppression of ACSL5 expression.

## Discussion

Cellular transformation relies on molecular signals that promote cell proliferation, obstruct cell death, inhibit cellular differentiation, and induce angiogenesis. Underlying these cellular processes, transformed cells also undergo significant metabolic adaptation [28-30]. In fact, many mutations important for the cancer phenotype control glucose metabolism.

We present a novel mechanism whereby cancer cells with oncogenic *KRAS *mutation, and expressing both HIF-1α and HIF-2α, can maximize ATP production and minimize ROS generation. The proposed mechanism is through the induction of enzymes important for mitochondrial cardiolipin synthesis. Cardiolipin is a mitochondria-specific phospholipid that intimately associates with numerous mitochondrial proteins, including complexes I-IV and F_1_F_0_ATP synthase. In this process, cardiolipin optimizes efficient electron transfer and maximizes ATP production, with minimal ROS generation.

The role of mitochondrial respiration in cancer has not been clearly elucidated. Two lines of evidence suggest that mitochondrial respiration might be important for cancer cells. First, cancer cells are metabolically active, which suggest that they may require efficient mitochondria. While some data have associated decreased tumorigenicity with metabolic conversion to mitochondrial respiration; other data have shown that the induction of mitochondrial biogenesis and respiration are associated with transformation [5,31]. For example, overexpression of the MYC oncogene has been shown to increase mitochondrial biogenesis and respiration [31]. Second, transformation by HRAS in human fibroblasts and bronchial epithelial cells not only increases glycolysis, but also increases mitochondrial respiration [5,32,33]. Our findings are consistent with these observations, suggesting that the induction of mitochondrial respiration contributes to carcinogenesis.

Our findings also have important clinical implications. It has recently been shown that therapies targeting the epidermal growth factor receptor (EGFR) provided clinical benefit in patients with head and neck, pancreatic, colorectal, and lung cancers. The mechanism of efficacy is through the inhibition of MAPK and PI3K/AKT signaling pathways, which are important for cell growth and cell survival, respectively. However, cancers with oncogenic *KRAS *mutations are primarily resistant, due to persistent signaling through these pathways [34,35]. Our data suggest that agents inhibiting both HIF-1α and HIF-2α, or their target genes, may likely be effective in treating cancers with oncogenic *KRAS *mutations.

To date, high-throughput small-compound screens have identified several classes of anticancer agents that disrupt HIF-1α function; including inhibition of its transcriptional activity, synthesis, or protein stability [7,36]. Due to the partial structural and functional similarities between HIF-1α and HIF-2α, some of the already identified HIF-1α inhibitors also inhibit HIF-2α. Our data suggest that it may be advantageous to further develop compounds that are effective at inhibiting both HIF-1α and HIF-2α for cancer treatment. As such, these isogenic HIF1α and HIF-2α knockout cell lines would provide invaluable tools for primary and secondary screens to systematically identify dual HIF-1α and HIF-2α inhibitors.

## Methods

### Cell lines

All cancer cell lines were acquired from the American Type Culture Collection (Manassas, VA). HCT116 and LOVO are human colon cancer cell lines. Isogenic cell lines with somatic disruption of oncogenic *KRAS*, *HIF-1α*, and *HIF-2α *genes were derived from HCT116 (HCT116^WT KRAS^, HCT116^HIF-1α-/-^, HCT116^HIF-2α-/-^, HCT116^HIF-1α-/-HIF-2α-/-^) cells as previously described [30]. Cells were grown in McCoy5A media, supplemented with 10% FBS and 1% penicillin/streptomycin (Invitrogen, Carlsbad, CA).

### Clonogenic survival assay

Cells were trypsinized, counted, and then seeded at low density (5000 cells per well) on six-well tissue culture plates and allowed to grow undisturbed at 37°C, in 5% CO_2 _for 10 days and then stained with crystal violet.

### Gene expression profiling

HCT116, HCT116^*HIF-1α-/-*^, HCT116^*HIF-2α-/-*^, HCT116^*HIF-1α-/-HIF-2α-/-*^, and HCT116^wt *K-RAS *^cells were seeded at low density 5000 cells per well on 6-well tissue culture plates and allowed to grow undisturbed at 37°C, in 5% CO_2 _for 10 days. Cells were then harvested and total RNA extracted. Gene expression analyses on the samples were performed at the University of Michigan Comprehensive Cancer Center Affymetrix Core Facility. Commercial high-density oligonucleotide arrays were used (GeneChip Human Genome U133A; Affymetrix, Inc., San Clara, CA), following protocols and methods developed by the supplier.

### Real-time reverse transcription (RT)-PCR analysis

Total RNA from cell lines or xenografts were extracted, treated with DNAse I, and reverse transcribed as previously described [37]. Real-time PCR reactions were performed in triplicate on RT-derived cDNA, and relative values calculated as previously described [37]. Student's paired t test was used to determine statistical significance between groups.

### Western blot analysis

Whole-cell protein extracts were prepared from cells, separated by electrophoresis, transferred to nitrocellulose membranes, and probed with antibodies as described previously [20]. Antibodies were obtained from BD Transduction Laboratories (San Jose, CA; mouse anti-human HIF-1α), Sigma (St. Louis, MO; α-tubulin), Novus Biologicals (Littleton, CO; rabbit anti-human HIF-2α and mouse anti-human ACSL5), Pierce (Rockford, IL; peroxidase-conjugated anti-rabbit antibody), and Jackson Immunoresearch Laboratories (West Grove, PA; peroxidase-conjugated anti-mouse antibody). Antibody dilutions were as recommended by the manufacturer.

### Site-directed mutagenesis and dual-luciferase assay

An 1883 bp fragment from the 5' untranslated region of the *ACSL5 *gene containing the hypoxia response element (HRE) indicating a putative HIF-α binding site was PCR amplified and subcloned into the minimal promoter Firefly luciferase reporter construct, pGL3pro (Promega, Madison, WI). The HRE site is denoted as a diamond in Figure 2E and is located at +2066 from transcriptional initiation. The resulting ACSL5-pGL3pro contruct was subjected to site-directed mutagenesis at the HRE site to generate the ACSL5(-HRE)-pGL3pro construct. For site-directed mutagenesis, the HRE site at position -18723 from translation start was mutated from CACGT to GGGGT using the Quickchange site-directed mutagenesis kit (Stratagene, La Jolla, CA). These three constructs (pGL3pro, ACSL5-pGL3pro, and ACSL5(-HRE)-pGL3pro) were separately co-transfected with CMV-Renilla luciferase reporter construct into cells using Lipofectamine as previously described [37]. Luciferase activity was measured using the Dual-Luciferase Reporter Assay System (Promega, Madison, WI). These studies were performed on cells grown on 6-well plates at 50-70% confluence. Student's paired t test was used to determine statistical significance between groups.

### Phosphatidyl choline and cardiolipin measurement

Phospholipids were measured by the methods of Folch et al., in collaboration with Lipomics Technologies, Inc. (West Sacramento, CA) [38]. Lipids from cells were extracted in the presence of internal standards using chloroform:methanol (2:1 v/v). Individual lipid classes within each extract were separated by liquid chromatography. Each lipid class was trans-esterified in 1% sulfuric acid in methanol in a sealed vial under a nitrogen atmosphere at 100°C for 45 min. The resulting fatty acid methyl esters were extracted from the mixture with hexane containing 0.05% butylated hydroxytoluene and prepared for gas chromatography by sealing the hexane extracts under nitrogen. Fatty acid methyl esters were separated and quantified by capillary gas chromatography equipped with a 30 m DB-88MS capillary column and a flame-ionization detector. Student's paired t test was used to determine statistical significance between groups.

### Cellular oxygen (O_2_) consumption assay

Cells were transferred to a 96-well O_2 _Biosensor plate (BD Biosciences, San Jose, CA), at a density of 250,000 cells per well. After two hours, fluorescence was measured at excitation/emission of 485 nm/630 nm. Student's paired t test was used to determine statistical significance between groups.

### Methylthiazoletetrazolium (MTT) reduction

Succinate dehydrogenase activity was measured by the reduction of methylthiazoletetrazolium (MTT) dye, using the CellTiter 96 Assay (Promega, Madison, WI). Student's paired t test was used to determine statistical significance between groups.

### ATP measurement

Cells were harvested and lysed by repeated freeze-thaw cycles. Intracellular ATP concentrations were measured using the ATP assay kit (Biomedical Research Service Center at State University of New York, Buffalo, NY). In the presence of ATP, the enzyme luciferase catalyzes the oxidation of luciferin with concomitant emission of yellow green light. Measurements were made on a luminometer and compared with a standard curve of ATP concentrations. Student's paired t test was used to determine statistical significance between groups.

### ROS measurement

For measurement of cellular ROS, cells were incubated with 5 μM CM-H_2_DCFDA (Invitrogen, Carlsbad, CA) for 1 hour at 37°C, then analyzed by flow cytometry with excitation and emission wavelengths, 488/525 nm. For measurement of mitochondrial ROS, cells were incubated with 5 μM MitoSOX Red (Invitrogen, Carlsbad, CA) for 15 minutes at 37°C, then analyzed by flow cytometry with excitation and emission wavelengths, 510/580 nm.

### Gene knockdown

The *ACSL5 *gene was suppressed using lentiviral shRNA clones from Open Biosystems repository (Huntsville, AL), which is made available by the RNAi Consortium. The constructs were tested to identify ones that can achieve the most efficient knockdown. Negative control was scramble shRNA cloned into the same vector (pLKO.1) (Addgene, Cambridge, MA). For generation of viral stocks, 293T cells were seeded on 100-mm dishes 1 day prior to transfection. Lentiviral constructs (3 μg) together with the lentiviral helper pHR'8.2dR and pCMV-VSV-G vectors (3 μg and 0.3 μg, respectively) were cotransfected into 293T cells by the FuGENE 6 Transfection Reagent according to manufacturer's protocol (Roche, Indianapolis, IN). The lentiviral supernatants were collected 48 hours after transfection and stored in aliquots at -80°C. Cancer cells grown in 6-well plate at subconfluence were transduced with 2 ml shRNA lentiviral supernatant in the presence of 8 μg/ml polybrene. The supernatant was replaced with growth medium after 1 day, and cells were selected with antibiotics on day 2 post-transduction.

## Conflict of interests statement

The authors declare that they have no competing interests.

## Authors' contributions

SC performed the cellular assays, molecular studies, and Western blots. CJ and JW carried out the microarray gene expression analysis and bioinformatics. MC provided patients' tumor samples with matched normal tissue. DD participated in the study design and performed the stastistical analysis. LD conceived the study, participated in study design, coordinated the experiments, and drafted the manuscript. All authors have read and approved the final manuscript.

## Supplementary Material

Additional file 1 **Confirmation of the disruption of the oncogenic *KRAS *allele in HCT116^WT KRAS ^cells**. Sequencing of genomic DNA at exon 2 of the *KRAS *gene showed parental HCT116 cells have both oncogenic mutant and wild-type alleles at codon 13, whereas HCT116^WT KRAS ^cells have only wild-type alleles. Click here for file

Additional file 2 **Confirmation of the disruption of *HIF-1α *and/or *HIF-2α *genes by homologous recombination**. Western blots using antibodies to HIF-1α and HIF-2α were done, with antibody to α-tubulin as loading control. c, clone. Click here for file

Additional file 3 **Testing of ACSL5 knockdown by four lentiviral shRNA clones in comparison to lentiviral clone carrying scramble shRNA**. ACSL5 mRNA level relative to β-actin was measured by real-time reverse transcription-PCR (n = 5). Bars, stdev. Clone H45551 was the most effective in suppressing ACSL5 expression. Click here for file
