# Supplementary figures and images for: Oncogenic KRAS modulates mitochondrial metabolism in human colon cancer cells by inducing HIF-1α and HIF-2α target genes
Source: Mol Cancer. 2010 Nov 13;9:293. doi: 10.1186/1476-4598-9-293 (PMC2999617; doi:10.1186/1476-4598-9-293)

## Slide 1
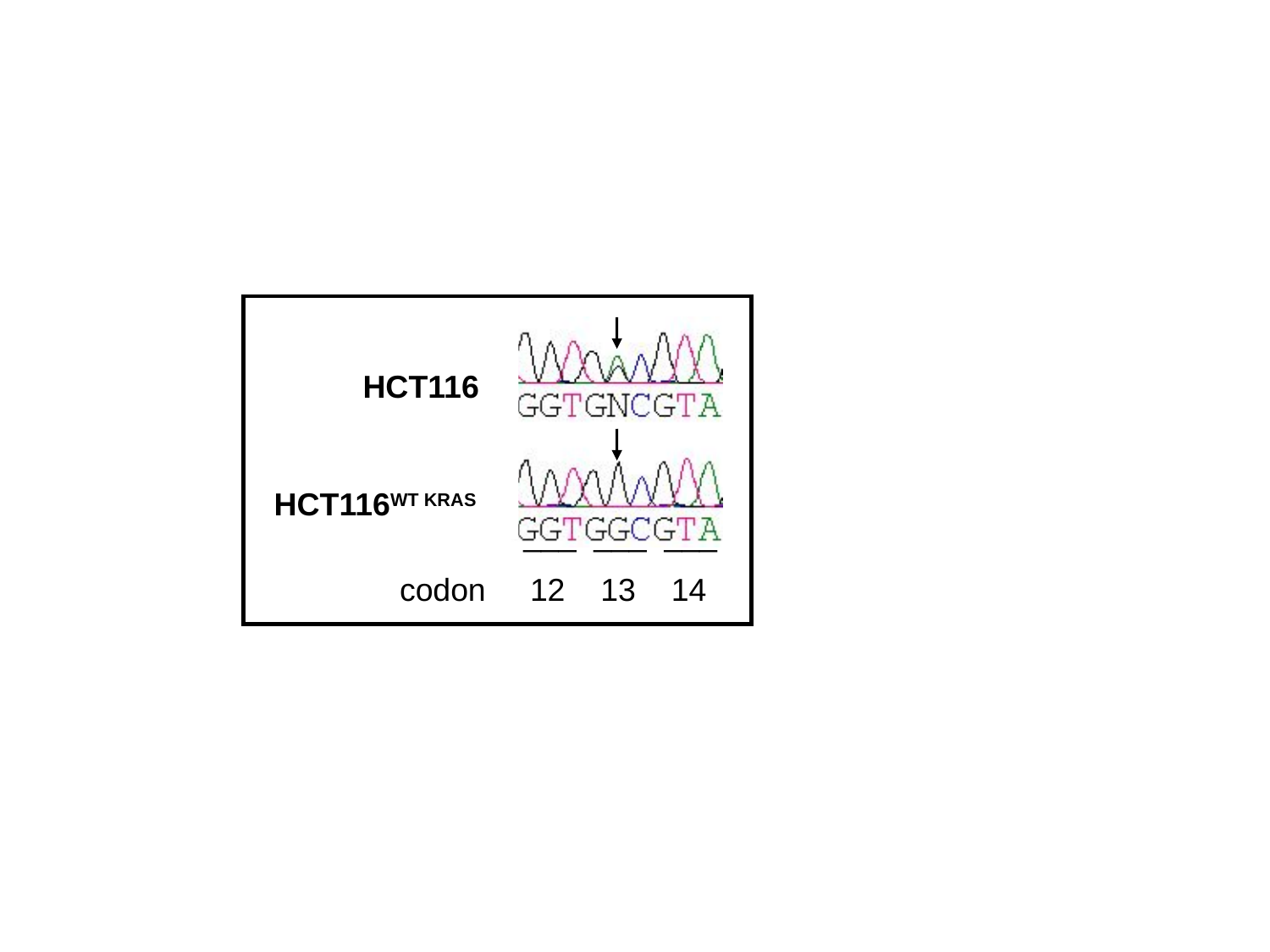

HCT116
HCT116WT KRAS
___ ___ ___
codon 12 13 14

Supplement: Additional file 1 — Confirmation of the disruption of the oncogenic KRAS allele in HCT116WT KRAS cells. Sequencing of genomic DNA at exon 2 of the KRAS gene showed parental HCT116 cells have both oncogenic mutant and wild-type alleles at codon 13, whereas HCT116WT KRAS cells have only wild-type alleles. [file 1476-4598-9-293-S1.PPT]

## Slide 1
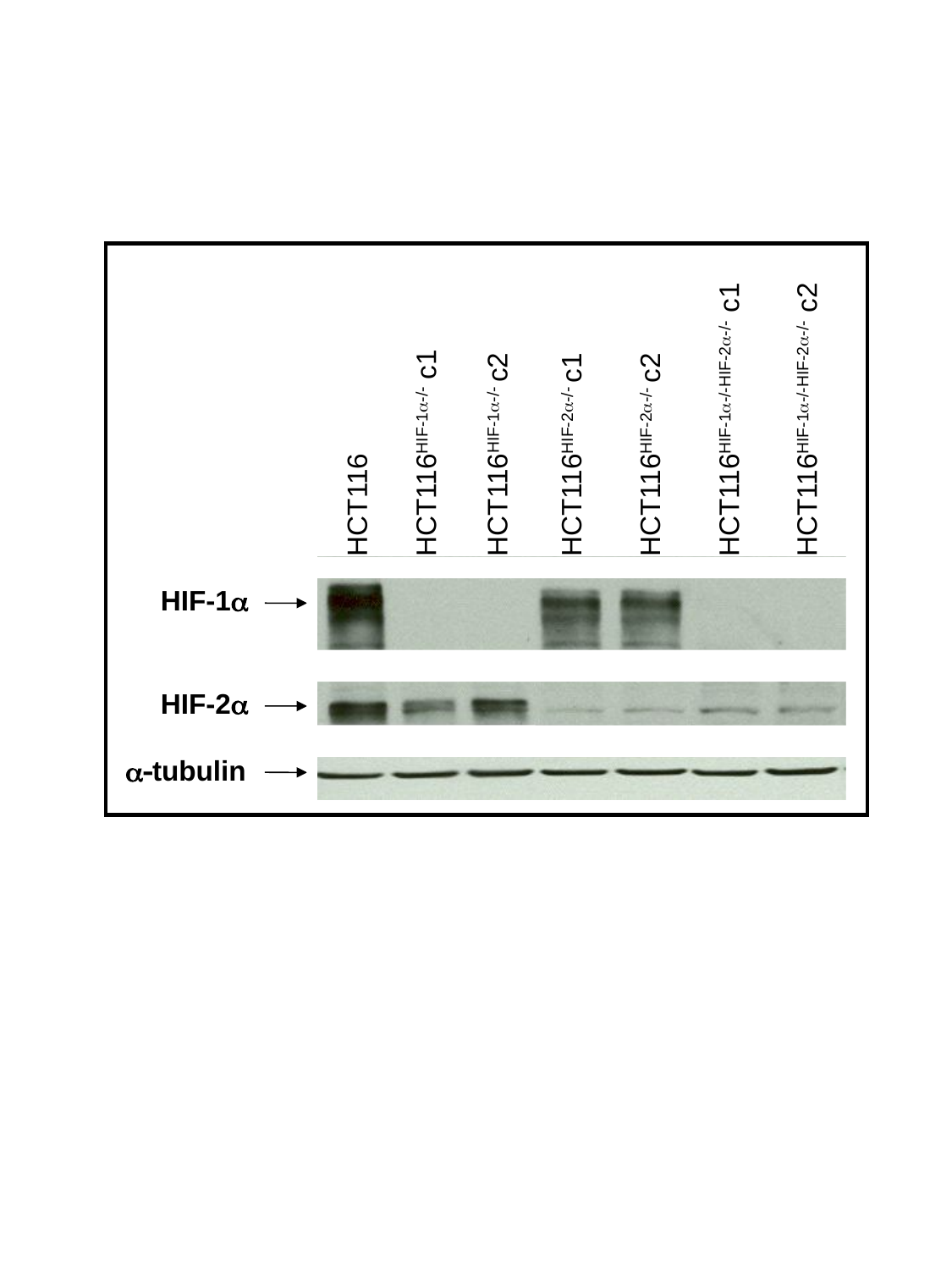

HCT116HIF-1α-/- c1
HCT116HIF-1α-/-HIF-2α-/- c1
HCT116HIF-1α-/-HIF-2α-/- c2
HCT116HIF-2α-/- c1
HCT116HIF-2α-/- c2
HCT116HIF-1α-/- c2
HCT116
HIF-1α
HIF-2α
α−tubulin

Supplement: Additional file 2 — Confirmation of the disruption of HIF-1α and/or HIF-2α genes by homologous recombination. Western blots using antibodies to HIF-1α and HIF-2α were done, with antibody to α-tubulin as loading control. c, clone. [file 1476-4598-9-293-S2.PPT]
