# Supplementary material for: Oncogenic KRAS modulates mitochondrial metabolism in human colon cancer cells by inducing HIF-1α and HIF-2α target genes
Source: Mol Cancer. 2010 Nov 13;9:293. doi: 10.1186/1476-4598-9-293 (PMC2999617; doi:10.1186/1476-4598-9-293)
Supplement: Additional file 3 — Testing of ACSL5 knockdown by four lentiviral shRNA clones in comparison to lentiviral clone carrying scramble shRNA. ACSL5 mRNA level relative to β-actin was measured by real-time reverse transcription-PCR (n = 5). Bars, stdev. Clone H45551 was the most effective in suppressing ACSL5 expression. [file 1476-4598-9-293-S3.PPT]

## Slide 1
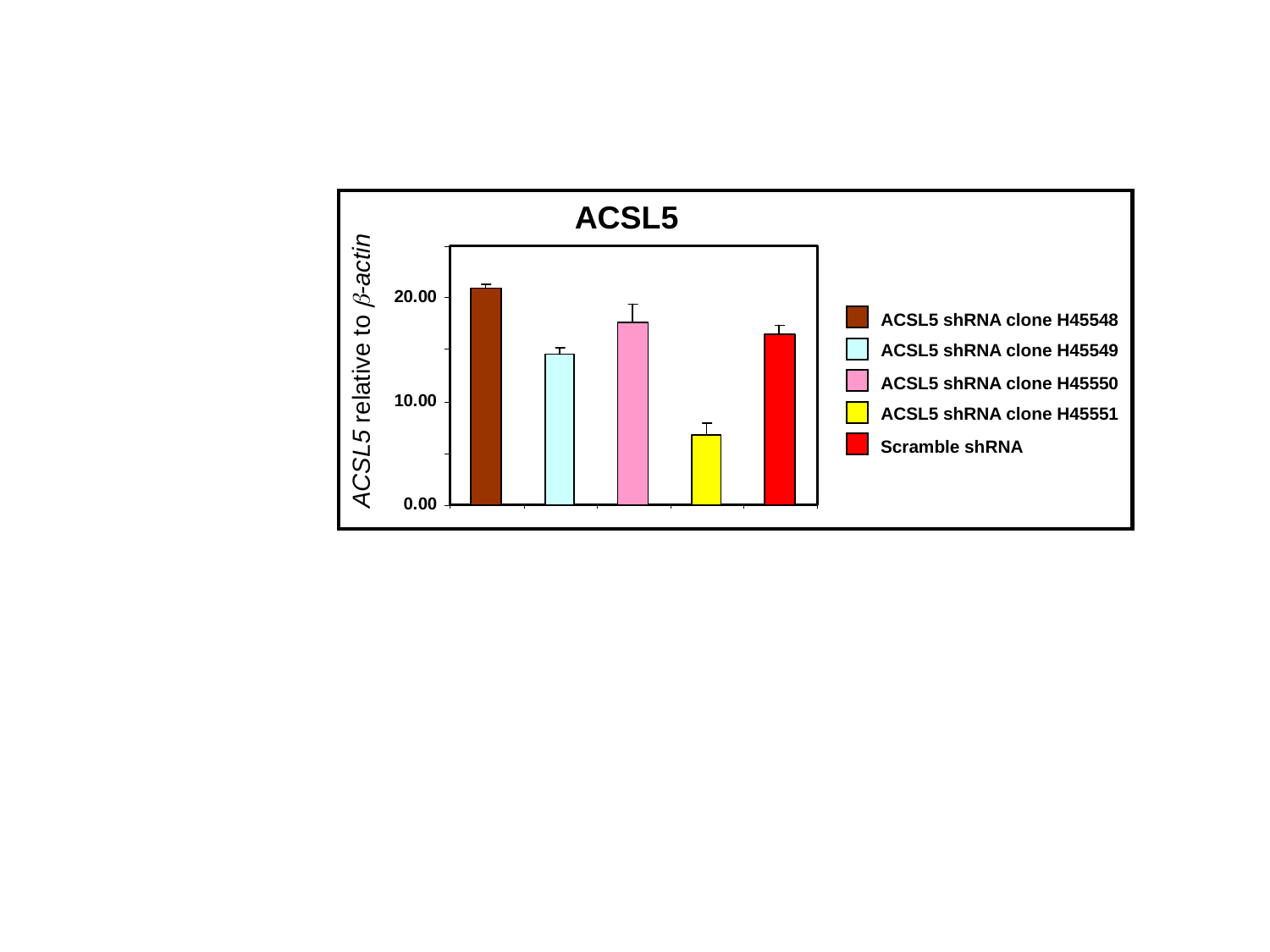

ACSL5
ACSL5 shRNA clone H45548
ACSL5 shRNA clone H45549
ACSL5 relative to β-actin
ACSL5 shRNA clone H45550
ACSL5 shRNA clone H45551
Scramble shRNA
| |
| --- |
| |
| --- |
